# Presence of Anti-MDA5 Antibody and Its Value for the Clinical Assessment in Patients With COVID-19: A Retrospective Cohort Study

**DOI:** 10.3389/fimmu.2021.791348

**Published:** 2021-12-20

**Authors:** Geng Wang, Qian Wang, Yeming Wang, Changzheng Liu, Linghang Wang, Hong Chen, Tao Jiao, Chaojun Hu, Xiaobo Lei, Li Guo, Lili Ren, Mengtao Li, Yan Zhao, Xiaofeng Zeng, Dingyu Zhang, Bin Cao, Jianwei Wang

**Affiliations:** ^1^ Department of Respiratory and Critical Care Medicine, West China Hospital, Sichuan University, Chengdu, China; ^2^ National Health Commission of the People's Republic of China (NHC), Key Laboratory of Systems Biology of Pathogens and Christophe Merieux Laboratory, Institute of Pathogen Biology, Chinese Academy of Medical Sciences and Peking Union Medical College, Beijing, China; ^3^ Department of Rheumatology, Peking Union Medical College Hospital, Peking Union Medical College & Chinese Academy of Medical Sciences, National Clinical Research Center for Dermatologic and Immunologic Diseases, Ministry of Science & Technology, Key Laboratory of Rheumatology and Clinical Immunology, Ministry of Education, Beijing, China; ^4^ Department of Pulmonary and Critical Care Medicine, Center of Respiratory Medicine, National Clinical Research Center for Respiratory Diseases, China-Japan Friendship Hospital, Beijing, China; ^5^ Institute of Respiratory Medicine, Chinese Academy of Medical Sciences, Peking Union Medical College, Beijing, China; ^6^ Department of Respiratory Medicine, Capital Medical University, Beijing, China; ^7^ Laboratory of Infectious Diseases Center of Beijing Ditan Hospital, Capital Medical University, Beijing, China; ^8^ The Second Affiliated Hospital of Harbin Medical University, Harbin, China; ^9^ Joint Laboratory of Infectious Diseases and Health, Wuhan Institute of Virology and Wuhan Jin Yin-Tan Hospital, China Academy of Sciences (CAS), Wuhan, China

**Keywords:** anti-melanoma differentiation-associated gene 5 (MDA5) antibody, COVID-19, dermatomyositis, acute respiratory distress syndrome (ARDS), innate immunity, autoimmune

## Abstract

**Background:**

Striking similarities have been found between coronavirus disease 2019 (COVID-19) and anti-melanoma differentiation-associated gene 5 (MDA5) antibody (Ab)-related dermatomyositis, implying a shared autoinflammatory aberrance. Herein, we aim to investigate whether the anti-MDA5 Ab is present in COVID-19 and correlates with the severity and adverse outcome of COVID-19 patients.

**Methods and Findings:**

We retrospectively recruited 274 adult inpatients with COVID-19 in this study, including 48, 164, and 62 cases of deaths, severe, and non-severe patients respectively. The anti-MDA5 Ab was determined by ELISA and verified by Western Blotting, which indicated that the positive rate of anti-MDA5 Ab in COVID-19 patients was 48.2% (132/274). The clinical and laboratory features, as well as outcomes between patients with positive and negative anti-MDA5 Ab were compared and we found that the anti-MDA5 Ab positive patients tended to represent severe disease (88.6% *vs* 66.9%, *P*<0.0001). We also demonstrated that the titer of anti-MDA5 Ab was significantly elevated in the non-survivals (5.95 ± 5.16 *vs* 8.22 ± 6.64, *P*=0.030) and the positive rate was also higher than that in the survivals (23.5% *vs* 12.0%, *P*=0.012). Regarding severe COVID-19 patients, we found that high titer of anti-MDA5 Ab (≥10.0 U/mL) was more prevalent in the non-survivals (31.2% *vs* 14.0%, *P*=0.006). Moreover, a dynamic analysis of anti-MDA5 Ab was conducted at different time-points of COVID-19, which revealed that early profiling of anti-MDA5 Ab could distinguish severe patients from those with non-severe ones.

**Conclusions:**

Anti-MDA5 Ab was prevalent in the COVID-19 patients and high titer of this antibody is correlated with severe disease and unfavorable outcomes.

## Introduction

Coronavirus Disease 2019 (COVID-19), caused by highly contagious severe acute respiratory syndrome coronavirus 2 (SARS-CoV-2), has become a pandemic involving more than 250 million cases globally by Nov 2021 (1). The average mortality is estimated to be 1% (2), but can raise up to 62% in critically ill patients, mostly due to acute respiratory distress syndrome (ARDS) (3). Therefore, early recognition of high risk COVID-19 patients has become an urgent task in this battle.

Accumulating evidence has demonstrated that high prevalence of anti-nuclear antibodies (35.6%) and lupus anti-coagulant (46.6%) were identified in hospitalized patients with COVID-19 (4). Thus, hypothesis that SARS-CoV-2 might trigger autoimmune aberrance in genetically predisposed subjects has been raised (5). Interestingly, striking similarities have been noted between multifaceted features of COVID-19 and a rare autoimmune disease, the anti-melanoma-differentiation-associated gene 5 (MDA5) antibody (Ab)-related dermatomyositis (DM) (6, 7). Both diseases can develop manifestations involving the lungs, skin (8, 9) and muscles (10). The initial radiological features of lung in anti-MDA5 Ab-related DM patients resemble severe and critical COVID-19 as well (11, 12). Furthermore, serum cytokine profiles are also similar in these two conditions, such as serum levels of ferritin, IL-6, IL-8, and IL-10 usually were elevated in patients with severe COVID-19 and rapid progressive interstitial lung disease (RP-ILD) secondary to anti-MDA5 Ab-related DM (13). The similarity of these two diseases implies shared underlying autoinflammatory/autoimmune mechanisms. To date, there is no report on whether anti-MDA5 Ab also exists in COVID-19 patients. It is well-known that MDA5 is a crucial cytoplasmic sensor for viral RNA and its expression is induced by RNA viruses. This activates the expression of antiviral type I and III interferons (IFNs) with inflammatory cytokines. Correspondingly, IFN signaling can induce the expression of MDA5 (14). SARS-CoV-2 infection has been reported to trigger the expression of MDA5 (15, 16). In addition, MDA5 is involved in pathogenesis of several autoimmune disorders as well (14), such as systemic lupus erythematosus (17, 18), multiple sclerosis (19), and even type 1 diabetes (20, 21). Nevertheless, it remains unclear whether the anti-MDA5 Ab plays a role in the pathophysiology of COVID-19 or whether it correlates with the disease severity. Some researchers have called for screening the anti-MDA5 Ab in severe COVID-19 patients (6, 7, 22).

In this study, we investigated the presence of anti-MDA5 Ab in patients with SARS-CoV-2 infection and to address its correlation with the clinical severity and outcomes of COVID-19.

## Methods

### Study Design, Setting, and Participants

This retrospective cohort study included three cohorts of adult patients (≥18 years old) from Jin Yin-Tan Hospital (Wuhan, China), Beijing Ditan Hospital (Beijing, China), and Heilongjiang Infectious Disease Hospital (Harbin, China), who were hospitalized from Dec 1, 2019 to Apr 19, 2020. All patients who were diagnosed with COVID-19 according to the Protocol for Control and Prevention of COVID-19 (Edition 7) promulgated by National Health Commission of China (23). All patients were followed until discharging from the hospitals or death of any cause.

**Figure 1:**
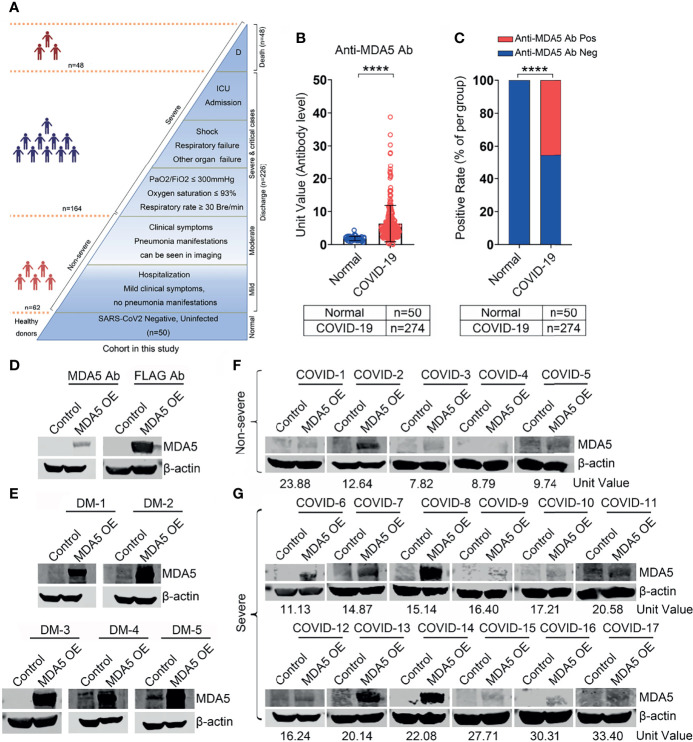
Anti-MDA5 Ab determined in patients with COVID-19. **(A),** Overview of the cohort in this study, including healthy donors (n=50) and COVID-19 patients (n=274). Of these patients, 62 non-severe COVID-19 patients included mild and moderate clinical performance, which was defined by the symptoms with or without mild lung change. Severe disease status (n=212): Clinical symptoms with severe lung change (Lesions progression> 50%), organ dysfunction, respiratory failure, shock, and intensive care unit (ICU) admission, and decease (n=48). **(B),** The titer of anti-MDA5 Ab was increased in patients with COVID-19. The plasma samples from healthy donors served as normal control (Normal). **(C),** Graph of positive rate of anti-MDA5 Ab in COVID-19 was higher than that in normal control (132/174, 48.2%). The numbers of normal control and COVID-19 patients are indicated underneath. *P* values were determined by using unpaired, two-sided Mann-Whitney *U*-test and χ^2^ test. *P* < 0.0001, ****. **(D–G),** MDA5 overexpression (OE) was achieved in 293-T cells and Western Blotting were performed with Anti-MDA5 Ab, Anti-FLAG Ab **(D)**, plasma form DM **(E)**, and plasma from COVID-19 patients **(F, G)**. β-actin is used as a loading control and the unit values from ELISA of each COVID-19 plasma samples are shown underneath.

A total of 274 patients were retrospectively recruited in this study, including 48, 164, and 62 cases of deaths, severe, and non-severe patients respectively (**Figure 1A** and **Table 1**). The median age was 56 years (IQR, 45-65 years), and 159 (58.0%) patients were male. The average disease course from onset of symptoms to discharge was 22.8 ± 9.6 days. According to the definition of disease severity, 212 (77.4%) patients were classified as severe disease. Nearly half of the patients (n=119, 43.4%) had underlying chronic diseases, including hypertension, coronary arterial disease, chronic lung disease, and diabetes mellitus. On admission, 43 (15.7%) patients were complicated with respiratory failure, shock or other organ dysfunctions. 31 (11.3%) were transferred to intensive care unit during their hospital stay, and 48 (17.5%) patients died. 134 (48.9%) out of 274 COVID-19 patients in our cohort had hypertension, diabetes, or cardiovascular disease, but no patients had autoimmune disease.

**Table 1:** Demographic, clinical, laboratory findings, and outcomes of patients with COVID-19.

	Total (n = 274)	Survivals (n = 226)	Non-survivals (n = 48)	*P*
**Demographic characteristics**				
Age, years	56 (45-65)	54 (44, 63)	64 (54-73)	**<0.001**
Sex				
Men	159 (58%)	125 (55%)	34 (71%)	-
Women	115 (42%)	101 (45%)	14 (29%)	0.054
Current smoker	16/188 (8%)	11/149 (7%)	5/39 (13%)	0.331
Chronic comorbidities (n, %)	134 (49%)	99 (44%)	35 (73)	**<0.001**
**Clinical symptoms**				
Fever	245 (89%)	199 (88%)	46 (96%)	0.128
Cough	218 (80%)	177 (78%)	41 (85%)	0.327
Fatigue	64 (23%)	46 (20%)	18 (38%)	**0.015**
Headache	20 (7%)	15 (7%)	5 (10%)	0.362
Dyspnea	46 (17%)	26 (12%)	20 (42%)	**<0.001**
Diarrhea	7 (3%)	6 (3%)	1 (2%)	1.000
Myalgia	47 (17%)	36 (16%)	11 (23%)	0.291
Skin Rash	0 (0%)	0 (0%)	0 (0%)	-
**Laboratory findings**				
Anti-MDA5-Ab titer, U/mL	4.86 (3.17-7.23)	4.64 (2.98-6.68)	5.91 (3.82-10.77)	**0.006**
White blood cell count, x10^9^ per L (n=215)	6.28 (4.48-8.91)	5.87 (4.37-7.92)	9.67 (7.57-12.01)	**<0.001**
Neutrophil count, x10^9^ per L (n=260)	4.67 (2.78-7.05)	3.89 (2.59-6.17)	8.29 (6.05-11.34)	**<0.001**
Lymphocyte count, x10^9^ per L (n=260)	0.91 (0.59-1.35)	0.99 (0.71-1.49)	0.53 (0.32-0.70)	**<0.001**
Hemoglobin, g/L (n=260)	124.0 (114.0-137.0)	125.0 (114.5-138.0)	123.0 (111.0-133.0)	0.250
Platelet count, x10^9^ per L (n=260)	208.5 (158.8-280.8)	220.0 (172.0-294.0)	158.0 (109.0-211.0)	**<0.001**
Albumin, g/L (n=260)	31.4 (28.5-35.9)	32.1 (29.6-36.9)	27.2 (25.3-30.5)	**<0.001**
Creatinine kinase, U/L (n=215)	77.0 (44.0-126.0)	61.0 (43.5-122.0)	96.5 (55.3-157.0)	**0.040**
Lactate dehydrogenase, U/L(n=215)	314.0 (236.0-430.0)	289.0 (224.5-364.5)	499.0 (405.0-632.3)	**<0.001**
D-dimer, mg/L (n=101)	0.97 (0.52-2.13)	0.90 (0.48-1.89)	2.21 (1.02-23.60)	**<0.001**
Brain natriuretic peptide, pg/mL (n=135)	34.7 (15.7-65.9)	30.7 (11.7-55.6)	69.3 (32.0-159.0)	**<0.001**
CRP, mg/L (n=196)	39.9 (14.6-105.0)	31.3 (12.3-79.8)	106.8 (55.7-160.0)	**<0.001**
IL-6 (n=167)	7.6 (5.8-10.9)	7.4 (5.7-9.3)	11.9 (7.2-16.1)	**<0.001**
Ferritin(ng/mL)(n=162)	703.4 (356.2-1324.6)	589.3 (321.9-1093.8)	1327.8 (909.0-2000.0)	**<0.001**
**Clinical severity and outcomes**				
Clinical severity				
Non-sever	62 (23%)	62 (27%)	0 (0%)	**-**
Severe	212 (77%)	164 (73%)	48 (100%)	**<0.001**
Other Organ dysfunction	43 (16%)	9 (4%)	34 (71%)	**<0.001**
ICU admission	31 (11%)	9 (4%)	22 (46%)	**<0.001**
Time from illness onset to hospital discharge, days (n=267)	24 (17-29)	24 (17-28)	24 (18-30)	0.358

Data are median (IQR), n (%), or n/N (%). p values p values comparing survivals and non-survivals are calculated by Mann-Whitney U test, χ² test, or Fisher’s exact test, as appropriate. COVID-19, coronavirus disease 2019; MDA5 melanoma differentiation-associated gene 5. BNP, brain natriuretic peptide; CRP, C-reactive protein; IL-6, interleukin-6; *χ² test comparing all subcategories. Bold values indicate that significant differences of demographics, clinical, laboratory findings, and outcomes between survivals and non survivals.

The plasma of patients with COVID-19 were collected within 24 hours after admission and stored at -80°C. The plasma of five patients with anti-MDA5 Ab-related DM were provided by the Biobank of Myositis Registry of Department of Rheumatology, Peking Union Medical College Hospital, Chinese Academy of Medical Sciences & Peking Union Medical College. All of the 5 DM patients were diagnosed based on the criteria of Bohan and Peter (24, 25).

### Data Collection

We extracted demographic, clinical, laboratory, treatment, and outcome data from medical and nursing records using standardized data collection forms (a revised version of case record form for severe acute respiratory infection shared by WHO and the International Severe Acute Respiratory and Emerging Infection Consortium). All data were checked by two investigators and a third researcher adjudicated any difference in interpretation between the two primary reviewers.

According to the clinical classification of COVID-19 by the Protocol for Control and Prevention of cases of COVID-19 (Edition 7) (23), we divided the patients into two groups on hospitalization: (1) Severe group, the patients fulfilled the diagnostic criteria of severe and critical cases, who meets any one of follows: i) Respiratory rate>30/min, ii) Pulse oxygen saturation<93%, iii) Oxygenation index<300 mmHg, or iv) respiratory failure or other organ dysfunction requiring transmission to intensive care unit. (2) Non-severe group, the patients’ severity was mild or moderate that didn’t meet the above criteria.

### ELISA and Western Blotting

IgG against MDA5 were detected in plasma samples using Anti-MDA5 ELISA Kit (Medical & Biological Laboratories Co., Ltd, Nagoya, Aichi, Japan), according to the manufacturer’s instructions. The unit value ≥5.0 U/mL is considered positive and the unit value ≥10.0 U/mL is defined as high titer of anti-MDA5 Ab.

The transfection of the plasmid expressing human MDA5 cDNA with a Flag tag was performed using Lipofectamine 2000 transfection reagent (Invitrogen) according to the manufacturer′s instruction. Western Blotting of proteins was performed as described previously (26). The antibodies used included those against Flag and MDA5 were purchased from Sigma-Aldrich Co. and the antibody against β-actin were obtained from Abcam Co.

Detailed experimental procedures are provided in the **Supplementary Methods**.

### Ethics Approval

The study was approved by the Institutional Review Board of Jin Yin-Tan Hospital (ChiCTR2000029308), and Infectious Disease Hospital of Heilongjiang Province (20200401). The requirement for informed consent was waived by the Ethics Commission of the designated hospitals for emerging infectious diseases as described previously.

### Statistical Analysis

For the detection of anti-MDA5 Ab, each experiment was repeated 3 times. Unpaired, two-sided Mann-Whitney *U*-test was performed to compare two groups unless otherwise indicated (χ^2^test). For the clinical analysis of anti-MDA5 Ab, descriptive statistics [percentages, means, standard deviations (SDs), medians, interquartile (IQR)] were provided for describe baseline demographic and clinical characteristics. The comparison of demographic, clinical, laboratory characteristics and outcomes across anti-MDA5 Ab positive/negative and survival/non-survival subgroups was performed by the Chi-squared tests or analysis of variance as appropriate. All statistical analyses were performed using SPSS 16.0 software (SPSS Inc., Chicago, IL, USA). *P*-values <0.05 were considered statistically significant.

## Results

### Anti-MDA5 Ab Is Identified in the Plasma of Patients With COVID-19

To determine the presence of anti-MDA5 Ab, ELISA analysis was employed to test the plasma collected from COVID-19 patients. We demonstrated that the titer of anti-MDA5 Ab is increased in the plasma of COVID-19 patients as compared with healthy controls (1.85 ± 0.67 *vs* 6.60 ± 5.50, *P*<0.0001) (**Figure 1B**). The plasma from five patients of anti-MDA5 Ab-related DM were used as positive controls (**Supplementary Figure 1**). The positive rate of anti-MDA5 Ab was also higher in COVID-19 patients than that in healthy controls (*P*<0.0001) (**Figure 1C**). These data were further validated by Western Blotting in selected COVID-19 plasma samples. To this aim, we firstly performed MDA5 overexpression in 293T cells as shown by Western Blotting analysis (**Figure 1D**). The plasma of anti-MDA5 Ab-related DM patients included in the ELISA were also confirmed (**Figure 1E**). Next, a total of 17 plasma samples of COVID-19 were subjected to Western Blotting analysis, which included five non-severe and 12 severe COVID-19 patients. Furthermore, we conducted Western Blotting in four healthy controls, the presence of MDA5 was not detected (**Supplementary Figure 4**). These findings showed that the anti-MDA5 Ab was detected in these examined samples as well (**Figures 1F, G**). Altogether, our data indicate that SARS-CoV-2 infection leads to an increased anti-MDA5 Ab titer in with COVID-19 patients.

### COVID-19 Patients With Positive Anti-MDA5 Ab Tend to Exhibit Severe Disease

**Figure 2:**
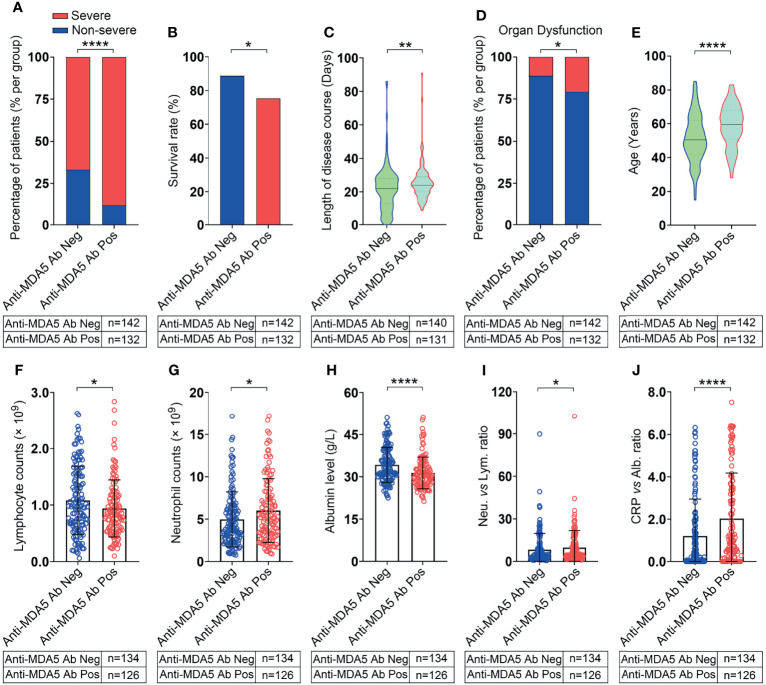
COVID-19 patients with positive anti-MDA5 Ab exhibit severe clinical performance. **(A),** Comparison of the percentage of COVID-19 patients with non-severe (mild & moderate) and severe performance in anti-MDA5 Ab negative (Anti-MDA5 Ab Neg) and anti-MDA5 Ab positive (Anti-MDA5 Ab Pos) group. **(B–J),** Comparison of clinical and demographic features of COVID-19 patients in anti-MDA5 Ab negative and positive groups. **(B),** survival rate; **(C),** total disease course; **(D)**, the percentage of organ dysfunction; **(E)**, age; **(F)**, lymphocyte number; **(G)**, neutrophils number; **(H)**, albumin levels; **(I),** the ratio of neutrophils versus lymphocytes (NLR); **(J)**, the ratio of C-reactive protein (CRP) versus albumin (CAR). The numbers of COVID-19 patients in each group are indicated underneath. *P* values were determined by using unpaired, two-sided Mann-Whitney *U*-test and χ^2^ test. *P* < 0.05, *; *P* < 0.01, **; *P* < 0.0001, ****.

The 274 recruited COVID-19 patients were stratified into two groups: the anti-MDA5 Ab negative group (<5.0 U/mL) and the positive group (≥5.0 U/mL) as the commercial kit suggested. The percentage of severe COVID-19 patients was much higher in the anti-MDA5 Ab positive group than that in the negative group (88.6% *vs* 66.9%, *P*<0.0001, χ^2^ test) (**Figure 2A**). The survival rate of the anti-MDA5 Ab positive group is much lower compared with the negative group (76.5% *vs* 88.0%, *P*=0.012, χ^2^ test) (**Figure 2B**). As expected, the COVID-19 patients with positive anti-MDA5 Ab tended to have much longer disease course at discharge and higher incidences of respiratory failure, shock and other organ dysfunction (**Figures 2C, D**).

A univariate analysis was employed to investigate the correlation between anti-MDA5 Ab and other COVID-19 prognostic factors (**Table 2**). We found that the titer of anti-MDA5 Ab was positively correlated with the age of COVID-19 patients (**Figure 2E**). We also noticed that COVID-19 patients with positive anti-MDA5 Ab depicted decreased lymphocytes and increased neutrophils (**Figures 2F, G**). The levels of albumin were found to decrease in anti-MDA5 Ab positive patients compared with the negative (**Figure 2H**). The ratio of neutrophils versus lymphocytes (NLR) and C-reactive protein (CRP) versus albumin (CAR) were much higher in anti-MDA5 Ab positive samples than that in the negative, indicating much severer inflammatory damage (**Figures 2I, J**). No significant difference was observed in Creatine Kinase (CK), lactate dehydrogenase (LDH), ferritin, and CRP (**Table 2**).

**Table 2:** Demographic, clinical, laboratory findings, and outcomes of patients with COVID-19.

	Anti-MDA5 Ab positive	Anti-MDA5 Ab negative	*P*
	(n = 132)	(n = 142)	
**Demographic characteristics**			
Age, years	59.0 (50.3, 68.0)	51.00 (42.00, 63.00)	**<0.001**
Sex	69 (52.3)	90 (63.4)	0.063
Men			
Women			
Current smoker	10 (9.8)	6 (7)	0.489
Chronic comorbidities (n, %)	75 (56.8)	59 (41.5)	**0.012**
**Clinical symptoms**			
Fever	120 (90.9)	125 (88.0)	0.439
Cough	117 (88.6)	101 (71.1)	**<0.001**
Fatigue	34 (25.8)	30 (21.1)	0.365
Headache	12 (9.1)	8 (5.6)	0.272
Dyspnea	25 (18.9)	21 (14.8)	0.358
Diarrhea	3 (2.3)	4 (2.8)	0.775
Myalgia	21 (15.9)	26 (18.3)	0.598
Skin Rash			
**Laboratory findings**			
Anti-MDA5-Ab titer, U/mL	7.36 (5.88, 11.04)	3.26 (2.23, 4.19)	**<0.001**
White blood cell count, x10^9^ per L (n=215)	6.9 (4.8, 9.4)	5.81 (4.3, 8.3)	0.079
Neutrophil count, x10^9^ per L (n=260)	5.17 (3.15, 7.90)	3.90 (2.58, 6.43)	**0.024**
Lymphocyte count, x10^9^ per L (n=260)	0.84 (0.57, 1.17)	0.98 (0.65, 1.54)	**0.040**
Hemoglobin, g/L (n=260)	123.00 (113.00, 136.25)	128.00 (115.75, 138.00)	0.193
Platelet count, x10^9^ per L (n=260)	209.50 (146.00, 285.25)	207.00 (169.50, 276.00)	0.754
Albumin, g/L (n=260)	30.45 (27.30, 33.50)	32.95 (29.65, 38.80)	**<0.001**
Creatinine kinase, U/L (n=215)	66.00 (43.50, 118.50)	73.50 (45.75, 158.75)	0.474
Lactate dehydrogenase, U/L (n=215)	323.00 (238.50, 447.00)	303.50 (227.00, 419.50)	0.410
D-dimer, mg/L (n=101)	0.85 (0.36, 1.92)	0.41 (0.00, 1.24)	**<0.001**
Brain natriuretic peptide, pg/mL (n=135)	36.60 (18.60, 81.30)	31.40 (10.00, 65.53)	0.107
CRP, mg/L (n=196)	42.70 (18.08, 110.30)	39.55 (10.30, 90.83)	0.095
IL-6 (n=167)	7.41 (5.53, 10.09)	8.02 (6.15, 11.39)	0.177
Ferritin (ng/mL) (n=162)	801.31 (381.88, 1397.92)	678.19 (320.31, 1251.58)	0.270
NLR (n=260)	5.26 (2.97, 12.07)	3.76 (2.24, 8.56)	**0.016**
PLR (n=260)	5.17 (3.15, 7.90)	3.90 (2.58, 6.43)	0.193
**Clinical severity and outcomes**			
Clinical severity	117 (88.6)	95 (66.9)	**<0.001**
Non-sever			
Severe			
Other Organ dysfunction	27 (20.5)	16 (11.3)	**0.037**
ICU admission	18 (13.6)	13 (9.2)	0.242
Death	31 (23.5)	17 (12.0)	**0.012**
Time from illness onset to hospital discharge, days (n=267)			

Data are median (IQR), n (%), or n/N (%). P-values were calculated by Mann-Whitney U test, χ² test, or Fisher’s exact test, as appropriate. COVID-19, coronavirus disease 2019; MDA5 melanoma differentiation-associated gene 5; BNP, brain natriuretic peptide;. CRP, C-reactive protein; IL-6, interleukin-6. χ² test comparing all subcategories. Bold values indicate that significant differences of demographics, clinical, laboratory findings, and outcomes between anti-MDA5 Ab positive and negative patients.

Taken together, our findings suggest that anti-MDA5 Ab is positively correlated with the clinical severity of COVID-19 patients.

### Correlation Between Anti-MDA5 Ab and COVID-19 Outcomes

**Figure 3:**
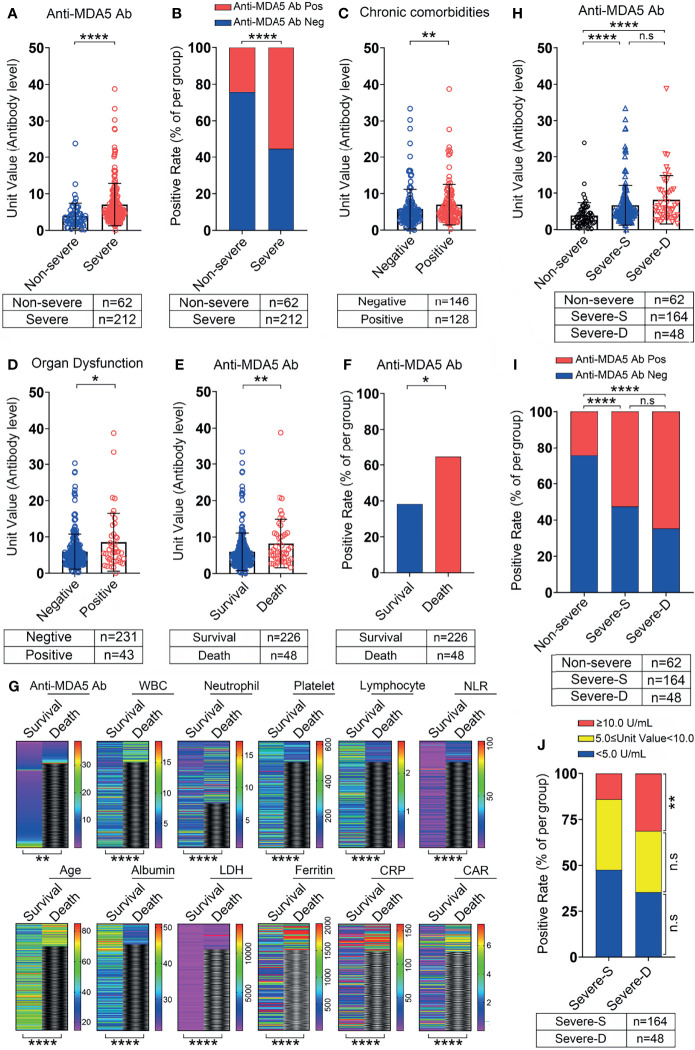
The correlation between anti-MDA5 Ab and the outcome of COVID-19 patients. **(A, B),** Comparison of the titer and positive rate of anti-MDA5 Ab in COVID-19 patients with non-severe and severe performance. **(C, D),** The titer of anti-MDA5 Ab in COVID-19 patients with or without chronic comorbidities **(C)** and organ failure **(D)**. **(E, F),** The titer **(E)** and positive rate **(F)** of anti-MDA5 Ab in the deceased patients with COVID-19. **(G),** Comparison of multiple variables in the survival and deceased patients with COVID-19 as shown in heatmap paragraphs. **(H, I),** The titer **(H)** and positive rate **(I)** of anti-MDA5 Ab in survival and dead patients with severe performance compared with that in the non-severe. **(J),** Comparison of the percentage of patients with high anti-MDA5 Ab (Unit value≥10.0 U/mL) in the survival and dead patients with severe performance. The numbers of COVID-19 patients in each group are indicated underneath. *P* values were determined by using unpaired, two-sided Mann-Whitney U-test and χ^2^ test. *P* < 0.05, *; *P* < 0.01, **; *P* < 0.0001, ****; ns, no significance.

The titer and positive rate of anti-MDA5 Ab were higher in severe COVID-19 patients as compared with the non-severe (**Figures 3A, B**). We also observed that the titer of anti-MDA5 Ab depicted a significant increase in COVID-19 patients with underlying chronic comorbidities, for instance, hypertension, diabetes, and cardiovascular disease (**Figure 3C**). An augment of this antibody was noticed in COVID-19 patients suffering from shock, respiratory or other organ failure (**Figure 3D**).

When comparing the level of anti-MDA5 Ab between the survival COVID-19 patients and the non-survivals, the titer of anti-MDA5 Ab was significantly upregulated in non-survivals (**Figure 3E**). Accordingly, its positive rate was higher in the non-survivals (**Figure 3F**). These data suggested that anti-MDA5 Ab had the potential to serve as a prognostic factor for COVID-19. Consistent with published predictive factors for COVID-19 outcomes, we found that the levels of LDH, ferritin, and CRP were significantly decreased in the non-survivals as compared with that in the survivals, and the number of lymphocytes was also markedly reduced in the non-survivals (**Figure 3G** and **Table 1**).

We further performed a comparison of the anti-MDA5 Ab in COVID-19 patients with non-severe, severe performance and those deceased. The titer of anti-MDA5 Ab and positive rate were increased in severe and deceased patients compared with the non-severe ones (**Figures 3H, I**). Although both of the titer and positive rate of anti-MDA5 Ab depicted a moderate increase in the deceased patients as compared to the severe ones, no significant difference was observed between these two clusters (**Figures 3H, I**). In addition, we addressed the difference between the survivals and non-survivals in severe COVID-19 patients using 2-fold cut-off value based on the ELISA kit and found that the percentage of COVID-19 patients with high titer of anti-MDA5 Ab (≥ 10.0 U/mL) was elevated in the non-survivals than that in the survivals (**Figure 3J**).

Altogether, our data indicate that anti-MDA5 Ab could be a marker for prognosis of COVID-19 patients and severe COVID-19 patients with high titer of anti-MDA5 Ab tend to have elevated mortality.

### Early Profile of Anti-MDA5 Ab Distinguishes the Prognosis of Non-Severe and Severe COVID-19

**Figure 4:**
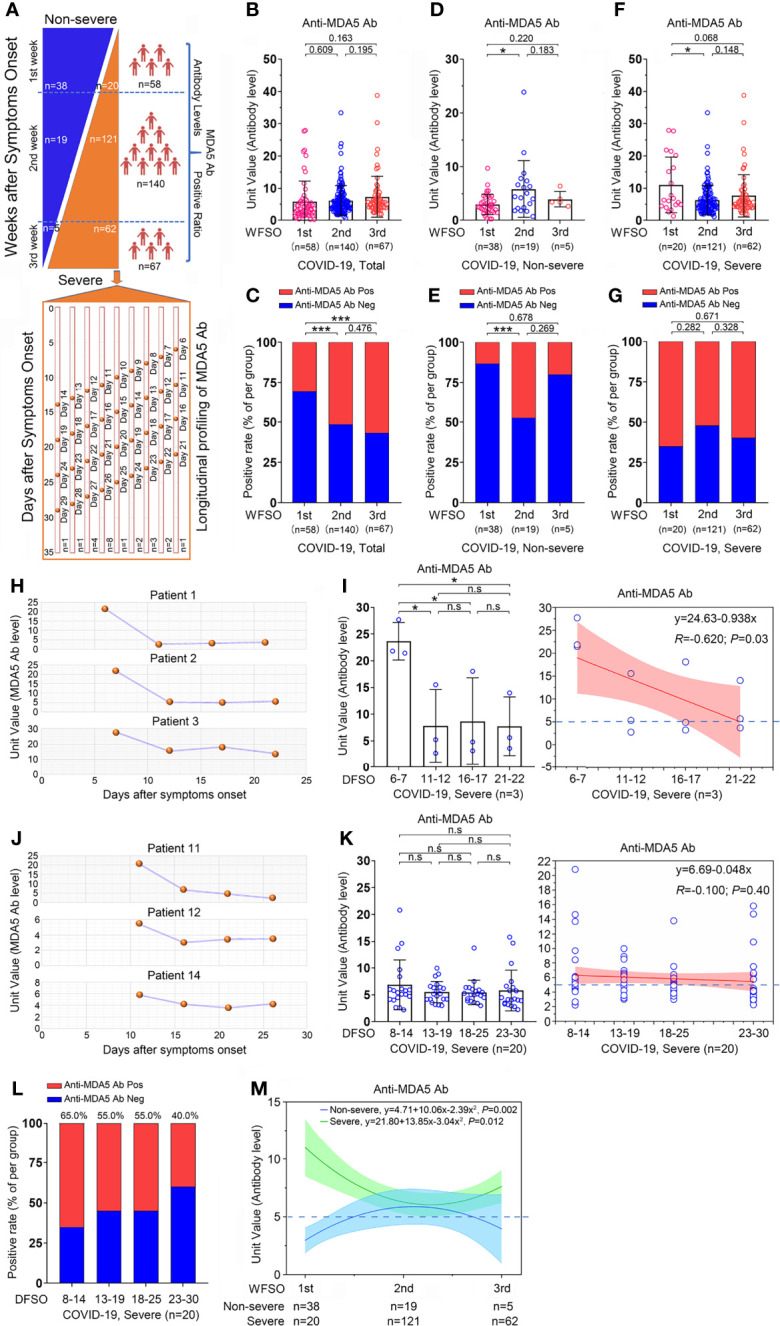
Overview of the anti-MDA5 profile in COVID-19. **(A),** Overview of the cross-sectional and longitudinal analyses in COVID-19. **(B, C),** A cross-sectional analysis of the anti-MDA5 Ab titer **(B)** and positive rate **(C)** is employed in 273 cases of COVID-19 patients. **(D, E),** A cross-sectional analysis of the anti-MDA5 Ab titer **(D)** and positive rate **(E)** is performed in non-severe COVID-19 patients. **(F, G),** A cross-sectional analysis of the anti-MDA5 Ab titer **(F)** and positive rate **(G)** is performed in severe COVID-19 patients. The samples were stratified into three clusters: WFSO-1, WFSO-2, and WFSO-3. The numbers of COVID-19 patients in each cluster are indicated underneath. *P* values were determined by using unpaired, two-sided Mann-Whitney U-test and χ^2^ test. *P* < 0.05, *; *P* < 0.001, ***. **(H, I),** A longitudinal analysis of anti-MDA5 Ab profiling in 3 patients **(H)**. 4 time-points were selected, which began from WFSO-1 (Patient 1, DFSO-6; Patient 2, 3, DFSO-7) [**(I)**, left panel]. Longitudinal data were also plotted over time continuously according to DFSO. Regression lines are indicated using the red solid line [**(I)**, right panel]. **(J, K),** A longitudinal analysis of anti-MDA5 Ab profiling in 20 patients. Of them, patient 11, 12, and 14 were shown in panel **(J)** 4 time-points were selected, which began from DWSO-2 [**(K)**, left panel]. Longitudinal data were also plotted over time continuously according to DFSO. Regression lines are indicated using the red solid line (**K**, right panel). **(L),** The positive rate of anti-MDA5 Ab determined in 20 patients as stated above at DFSO 8-14, 13-19, 18-25, and 23-30. **(M),** The cross-sectional data was also plotted according to days following symptom onset. Regression lines are indicated by the blue (non-severe) or green (severe) solid lines. The numbers of COVID-19 patients in each cluster are indicated underneath. *P* values were determined by using unpaired, two-sided Mann-Whitney U-test and χ^2^ test. *P* < 0.05, *; *P* < 0.001, ***; ns, no significance.

Since the alteration of anti-MDA5 Ab titer is correlated with the activity and outcome of DM, we asked whether the change of anti-MDA5 Ab was associated with the clinical features of COVID-19. To this end, a cross-sectional analysis was employed using the titer of anti-MDA5 Ab achieved from the whole disease course. Among the 274 recruited cases, one patient was lack of disease onset date and another 8 samples was collected more than 3 weeks after the onset date, we therefore stratified the 265 eligible cases into three clusters based on the weeks following symptoms onset (WFSO) and shown as WFSO-1, WFSO-2, and WFSO-3 (**Figure 4A**). A significant increase of the positive rate of anti-MDA5 Ab was observed in the samples from WFSO-2 and WFSO-3, as compared with WFSO-1, although no difference of the anti-MDA5 Ab titer was noticed in these three clusters (**Figures 4B, C**). These data suggest that the dynamic alteration of anti-MDA5 Ab might be various in the disease course of COVID-19 patients with diverse clinical performance. To test this idea, we compared anti-MDA5 Ab at three intervals as stated above in non-severe and severe patients, respectively. Interestingly, the titer of anti-MDA5 Ab in non-severe patients with COVID-19 was significantly increased at WFSO-2 as compared with that in WFSO-1, and then decreased at WFSO-3 (**Figure 4D**). Similar result was found in the positive rate of anti-MDA5 Ab (**Figure 4E**). In contrast, COVID-19 patients with severe performance exhibited high titer of anti-MDA5 Ab at the disease onset (WFSO-1) and then decreased at WFSO-2 and -3 (**Figure 4F**). However, no significant alteration was observed in the positive rate of this antibody at all three intervals, indicating that high titer of anti-MDA5 was preserved in the disease course of severe COVID-19 (**Figure 4G**).

We further determined the titer of anti-MDA5 Ab in sequential samples from severe COVID-19 patients as shown in **Figure 4A**. The titer of anti-MDA5 Ab in patient #1, #2, and #3 depicted a similar alteration compared with that in the cross-sectional analysis (**Figures 4H, I**). Next, the titer of anti-MDA5 Ab was examined in the samples collected at the WFSO-2, that is, the days following symptoms onset (DFSO) 8-14. We found that the titer and positive rate of anti-MDA5 Ab remained substantial (**Figures 4J**–**L** and **Supplementary Figure 2**).

Collectively, our data indicate that COVID-19 patients with high titer of anti-MDA5 Ab initially tend to develop severe disease.

## Discussion

The present study, for the first time, identified and confirmed the prevalence of anti-MDA5 Ab in COVID-19 patients by both ELISA and Western blots. We also demonstrated that the positive rate and titer of anti-MDA5 Ab was associated with the clinical severity and outcomes of COVID-19. In severe COVID-19 patients, we found that high titer of anti-MDA5 Ab (≥10.0 U/mL) was more prevalent in non-survival patients. Moreover, early profile of anti-MDA5 Ab could distinguish severe patients from non-severe ones. Our study provides the evidence that early screening of anti-MDA5 Ab might help identify high risk population and predict the outcome of patients with COVID-19.

MDA5 is a crucial antiviral factor and has been previously reported to involve in SARS-CoV, MERS-CoV, and SARS-CoV-2 infections (15, 27, 28). Interestingly, MDA5 is also involved in several autoimmune disorders such as anti-MDA5 Ab-related DM. Therefore, it is not surprising that COVID-19 and anti-MDA5 Ab-related DM share similar features of hyperinflammation and multi-systemic manifestations, especially RP-ILD that results in ARDS and death. In this study, we determined anti-MDA5 Ab in as many as 48.2% patients with COVID-19. Our study revealed a positive correlation between the anti-MDA5 Ab and the severity of COVID-19, and high titer of anti-MDA5 Ab was associated with higher mortality in severe COVID-19 patients. Similar observation was reported in anti-MDA5 Ab-related DM patients (29). However, the titer of this antibody is even higher in anti-MDA5 Ab-related DM than that in COVID-19. This may indicate that high titer of anti-MDA5 Ab probably is related to an uncontrolled autoinflammation and autoimmune response to SARS-CoV-2 infection in genetically predisposed hosts. Furthermore, our study also demonstrated that elder age, chronic comorbidities, lymphocytopenia, hypoalbuminemia, hyperferritinemia, increased D-dimer and CRP levels were more prevalent in COVID-19 patients with organ dysfunction and the mortality was comparatively high, which has been reported in previous studies and implies a dysregulation of inflammation (11, 30–33).

It is well known that an early innate immune response in host cells, mediated by pattern recognition receptors (PRRs) and the type I and III interferon (IFN) system, is crucial for the control of SARS-CoV-2 infection (34). In addition, higher basal expression of MDA5 is triggered by SARS-CoV-2 infection (35). It suggests that the increased MDA5 expression may represent a protective role against SARS-CoV-2 infection. However, the function and the underlying mechanism of anti-MDA5 Ab remains unclear in the pathological process of SARS-CoV-2 infection or anti-MDA5 Ab-related dermatomyositis. We speculate that SARS-CoV-2 infection may result in the leak of MDA5 from infected cells, and the production of abnormal autoantibodies may mediate immune damage. We had detected IFN-γ in part of our patients, there were no significant correlation between the level of anti-MDA5 Ab and that of IFN-γ (Data not shown). Unfortunately, we were not able to measure MDA5 and type I and III interferon levels in this study due to the difficulty to obtain sufficient amount of blood samples during the early stage of SARS-CoV-2 outbreak.

It has been reported that the change of anti-MDA5 Ab titer correlates with disease activity and predicts treatment response and disease outcome in patients with DM and rapidly progressive interstitial lung disease (29). Our data also indicated that the dynamic alteration of anti-MDA5 Ab clearly varied in COVID-19 patients with diverse clinical severities. In the non-severe patients, the titer of anti-MDA5 Ab is upregulated in week 2 after symptom onset and then decreased, suggesting that the IFNs-MDA5 circuit is under fine-tuning regulation and the immune homeostasis is preserved in the total process of SARS-CoV-2 infection (**Figure 4M**). However, in the severe COVID-19 patients, the titer of anti-MDA5 Ab boosts up in the 1st week after symptom onset and subsequently remains at a high positivity although a decreased titer is observed at weeks 2, 3, and 4 (**Figure 4M**). These data further supported that the MDA5 signaling might be persistently over-activated in severe COVID-19 patients. These findings also suggest that early screening and serially monitoring of anti-MDA5 Ab titer has the potential to predict the disease progression of COVID-19.

Several studies have already shown effectiveness of tocilizumab (IL-6 receptor blockade) (36), ruxolitinib (JAK inhibitor) (37) and tacrolimus (38) in inhibiting SARS-CoV-2 replication, improving the chest tomography or facilitating clinical improvement. Recently, dexamethasone has also been reported to improve the survival in severe COVID-19 patients as well (39). Our findings provide supportive evidence that anti-inflammation and immunosuppressive therapy might be compromising strategy for the treatment of COVID-19, especially in those with high titer of anti-MDA5 Ab.

There are several limitations in our study. Firstly, since MDA5 is validated as a general sensor for diverse RNA viruses, no evidence has addressed whether anti-MDA5 Ab is present in the infection of other RNA viruses, for instance, influenza virus, enterovirus, and other coronaviruses. Therefore, the specificity of anti-MDA5 Ab in COVID-19 need to be further investigated. Secondly, in our study, we detected anti-MDA5 Ab in limited number (274) of COVID-19 patients and we could not obtain sufficient consecutive samples due to limited supportive resources in the early stage of SARS-CoV-2 outbreak. For the same reason, we did not measure the dynamic variation of anti-MDA5 Ab at different days within the first week following the disease onset, which is crucial to illustrate the generation course of anti-MDA5 Ab. Due to the limited sampling, we were not able to further evaluate whether anti-MDA5 Ab is an independent predictive factor for the death in COVID-19 or could be included in a risk stratification model. Thirdly, all patients were from China and it is not clear whether patients with other genetic backgrounds would have same results. Our findings are to be validated in a larger population of different ethnicities in future.

## Conclusions

Overall, we, for the first time, revealed that anti-MDA5 Ab is present in patients with COVID-19 and correlates with severe disease and poor outcomes. Early screening and serially monitoring of anti-MDA5 Ab titer have the potential to predict the disease progression of COVID-19.

## Data Availability Statement

The original contributions presented in the study are included in the article/**Supplementary Material**. Further inquiries can be directed to the corresponding authors.

## Ethics Statement

The study was approved by the Research Ethics Committee of the participating hospitals and the ethical board of the Institute of Pathogen Biology, Chinese Academy of Medical Sciences. The requirement for informed consent was waived by the Ethics Commission of the designated hospitals for emerging infectious diseases as described previously. The patients/participants provided their written informed consent to participate in this study.

## Author Contributions

CL, QW, YW, GW, LW, and HC contributed equally to this paper. CL, QW, BC and JW conceived and designed the study. CL, QW, GW, YW, LW, and HC contributed to data collection, data analysis, and data interpretation. CL, GW, and TJ performed the experiments. CH, XL, LG, LR, ML, YZ, and XZ contributed to literature search and data collection. CL, QW, BC, and JW drafted the manuscript. The corresponding author attests that all listed authors meet authorship criteria and that no others meeting the criteria have been omitted. All authors approved the final version of the manuscript.

## Funding

National Key R&D Program of China (2020YFA0707600) and Chinese Academy of Medical Sciences Innovation Fund for Medical Sciences (CIFMS 2018-I2M-1-003, 2019-I2M-1-006, 2019-I2M-2-008), National Science Grant for Distinguished Young Scholars (81425001/H0104), The Beijing Science and Technology Project (D151100002115004), The National Natural Science Foundation of China (81930063), Beijing Municipal Science and Technology Commission Program (Z191100006619102).

## Conflict of Interest

The authors declare that the research was conducted in the absence of any commercial or financial relationships that could be construed as a potential conflict of interest.

## Publisher’s Note

All claims expressed in this article are solely those of the authors and do not necessarily represent those of their affiliated organizations, or those of the publisher, the editors and the reviewers. Any product that may be evaluated in this article, or claim that may be made by its manufacturer, is not guaranteed or endorsed by the publisher.
