# Supplementary figures and images for: Presence of Anti-MDA5 Antibody and Its Value for the Clinical Assessment in Patients With COVID-19: A Retrospective Cohort Study
Source: Front Immunol. 2021 Dec 20;12:791348. doi: 10.3389/fimmu.2021.791348 (PMC8720853; doi:10.3389/fimmu.2021.791348)

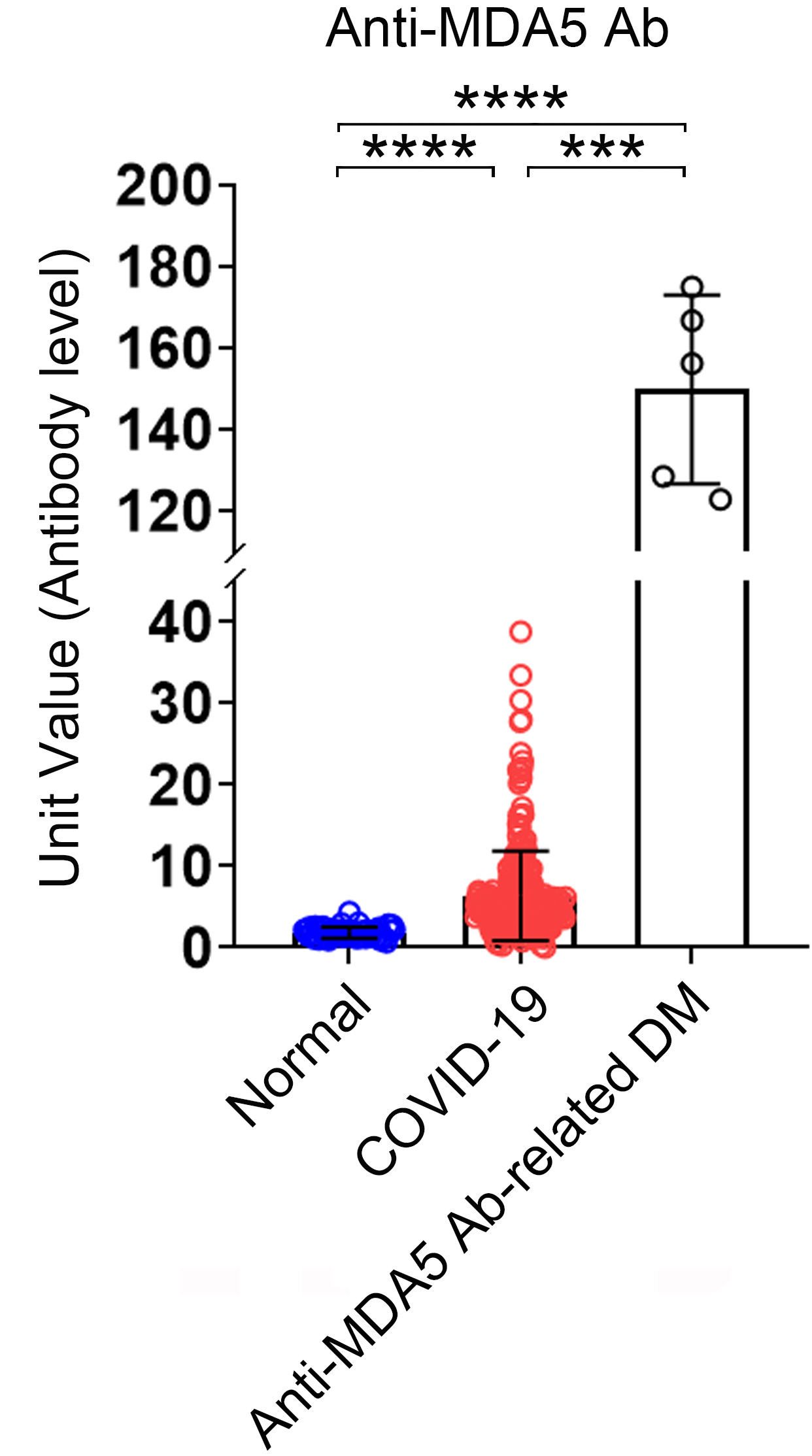

Supplement: Supplementary file 1 [file Image_1.jpeg]

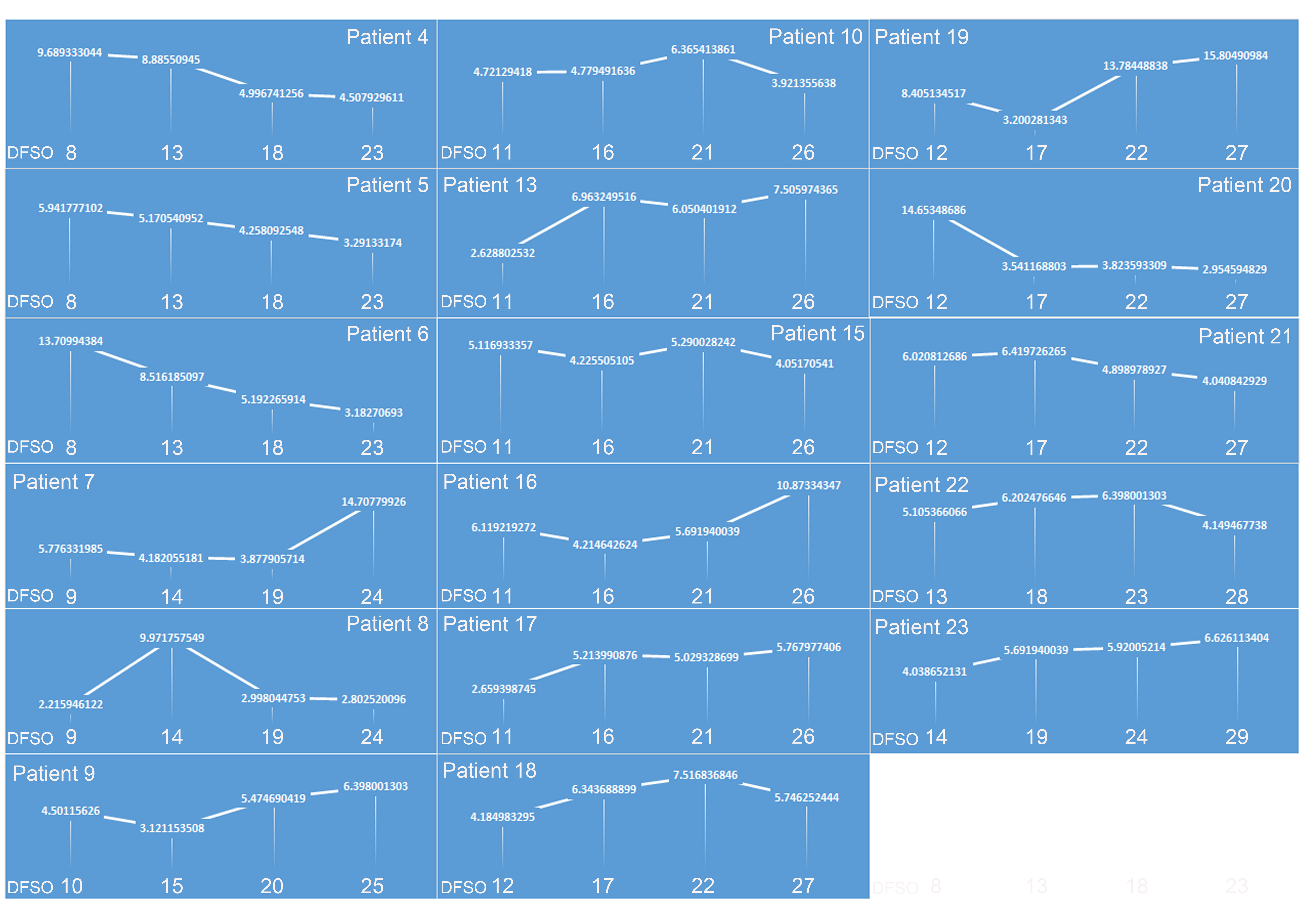

Supplement: Supplementary file 2 [file Image_2.jpeg]

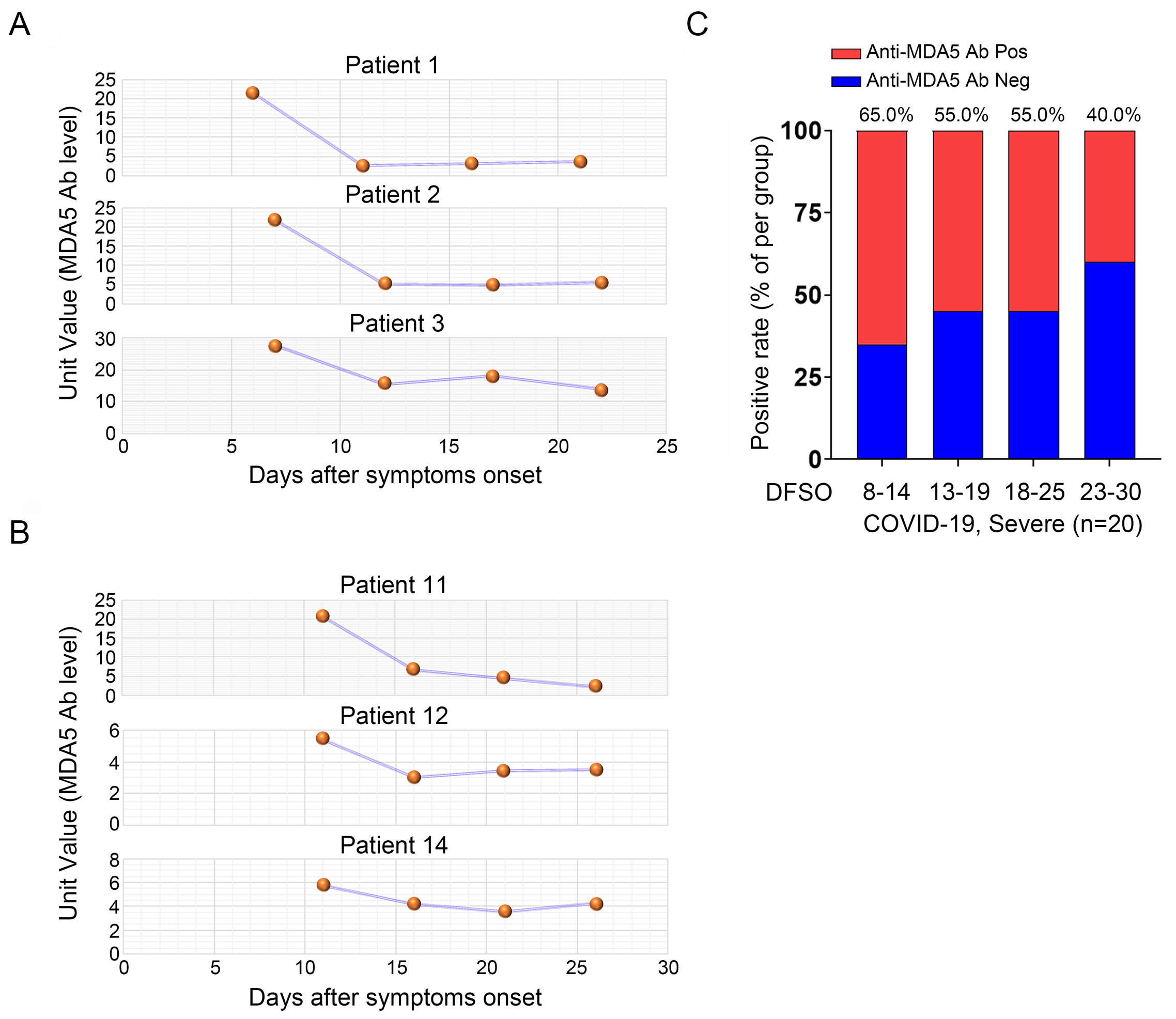

Supplement: Supplementary file 3 [file Image_3.jpeg]

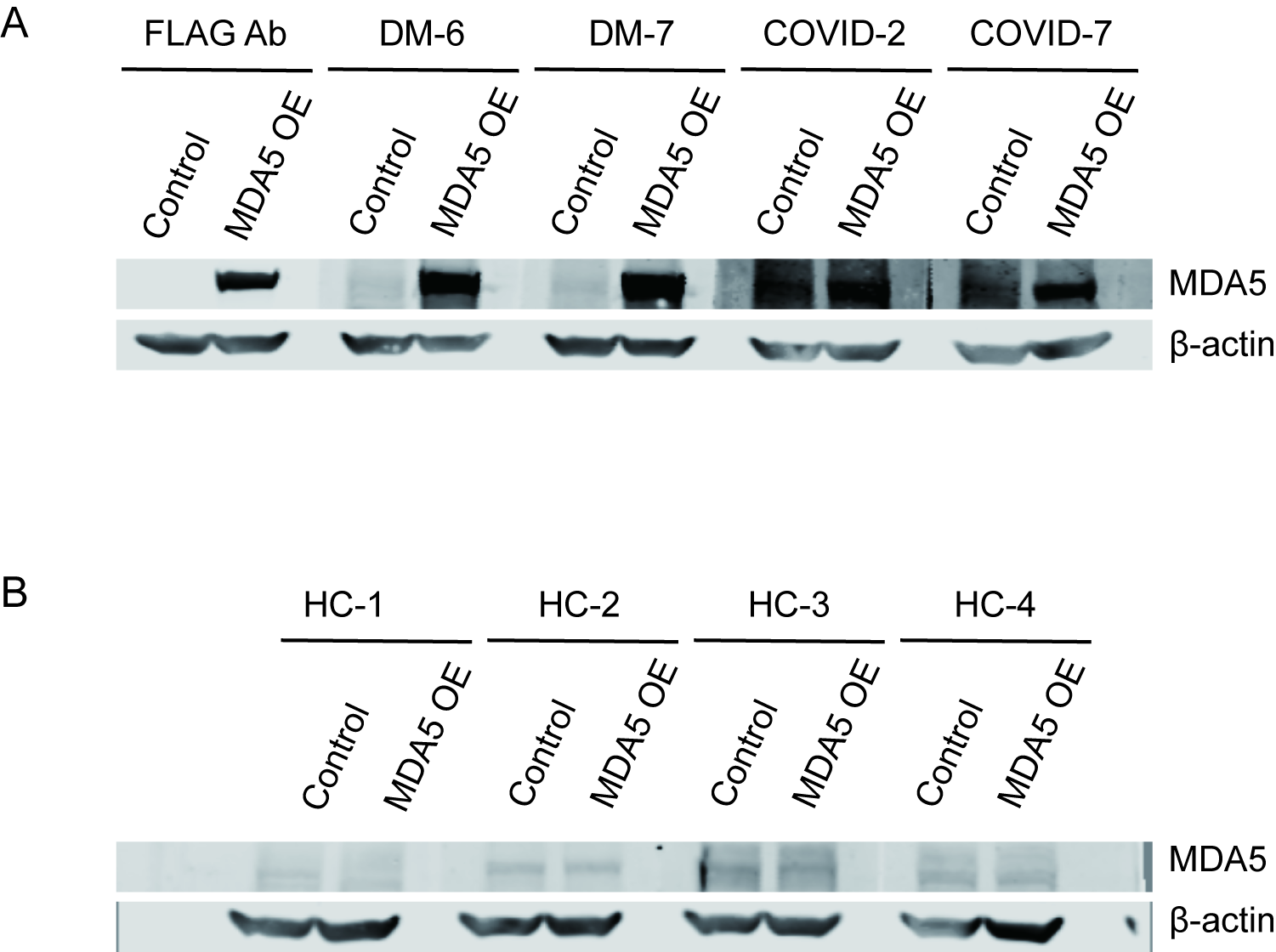

Supplement: Supplementary file 4 [file Image_4.tif]
